# Readiness of hospitals to provide Kangaroo Mother Care (KMC) and documentation of KMC service delivery: Analysis of Malawi 2014 Emergency Obstetric and Newborn Care (EmONC) survey data

**DOI:** 10.7189/jogh.07.020802

**Published:** 2017-12

**Authors:** Kondwani Chavula, Dyson Likomwa, Bina Valsangkar, Richard Luhanga, Lydia Chimtembo, Queen Dube, Wasihun Andualem Gobezie, Tanya Guenther

**Affiliations:** 1 Save the Children International, Lilongwe, Malawi; 2 Saving Newborn Lives, Save the Children, Washington, D.C., USA; 3 University of Malawi, College of Medicine & Ministry of Health, Blantyre, Malawi; 4 Averting maternal death and disability (AMDD), Ethiopia

## Abstract

**Background:**

Malawi introduced Kangaroo Mother Care (KMC) in 1999 as part of its efforts to address newborn morbidity and mortality and has continued to expand KMC services across the country. Yet, data on availability of KMC services and routine service provision are limited.

**Methods:**

Data from the 2014 Emergency Obstetric Newborn Care (EmONC) survey, which was a census of all 87 hospitals in Malawi, were analyzed. The WHO service availability and readiness domains were used to generate indicators for KMC service readiness and an additional domain for documentation of KMC services was included. Levels of KMC service delivery were quantified using data extracted from a 12–month register review and a KMC initiation rate was calculated for each facility by dividing the reported number of babies initiated on KMC by the number of live births at facility. We defined three levels of KMC readiness and two levels of KMC operational status.

**Results:**

79% of hospitals (69/87) reported providing inpatient KMC services. More than half of the hospitals (62%; 54/87) met the most basic definition of readiness (staff, space for KMC and functional weighing scale) and 35% (30/87) met an expanded definition of readiness (guidelines, staff, space, scale and register in use). Only 15**%** (13/87) of hospitals had all KMC tracer items. Less than half of the hospitals (43%; 37/87) met criteria for KMC operational status at minimum levels (≥1/100 live births), and just 16% (14/87) met criteria for KMC operational status at routine levels (≥5/100 live births).

**Conclusions:**

Our study found large differences between reported levels of KMC services and documented levels of KMC readiness and service provision among hospitals in Malawi. It is recommended that facility assessments of services such as KMC include record reviews to better estimate service availability and delivery. Further efforts to strengthen the capacity of Malawian hospitals to deliver KMC are needed.

Preterm birth is one of the leading causes of newborn morbidity and mortality globally [1–3]. Malawi has one of the highest rates of preterm births in the world, with an estimated 18% of all live births occurring before 37 completed weeks of gestation [2]. Kangaroo Mother Care (KMC) is strongly recommended by the World Health Organization (WHO) for the routine care of stable newborns weighing ≤2000 g as soon as they are clinically stable as an evidence–based intervention to improve preterm birth outcomes [4]. Kangaroo Mother Care is defined by WHO as early, continuous and prolonged skin–to–skin contact between the mother (or other caregiver) and the baby, and exclusive breastfeeding (ideally) or feeding with expressed breastmilk [4].

Malawi was an early adopter of KMC, introducing the intervention on a pilot basis in 1999 as part of its efforts to address newborn morbidity and mortality [5]. In 2005, KMC was integrated into national policy as routine care of preterm and low birth weight (LBW) babies. During the same period there was adoption of the Malawi National Guidelines on KMC [6] and incorporation of KMC into the Ministry of Health (MoH) workplan for 2005/6. The KMC guidelines were revised in 2009 to incorporate guidelines for ambulatory and community KMC [7] and KMC was integrated into the Sexual and Reproductive Health and Rights programs [8]. Malawi continued the expansion of KMC services across the country and by 2011, KMC was reportedly established in all central– and district–level hospitals as well as several first–level health facilities [5].

In July 2015, Malawi launched its Every Newborn Action Plan (ENAP), which aims to bring partners together to accelerate progress towards ending preventable newborns deaths. The major goal of the Malawi ENAP is to achieve equitable and high–level coverage of quality essential interventions and commodities for maternal and newborn health and ultimately halving the NMR to 15 per 1000 live births by 2035 [9]. High–impact, cost–effective interventions for newborn health, like breastfeeding support and KMC, form one component of integrated health services for newborn health. Within its ENAP plan, Malawi has established a target that 75 percent of eligible preterm and low birth weight newborns should be managed with facility–based KMC by 2020 and 90 percent by 2035 [9].

Despite KMC being national policy in Malawi for the last decade, data on availability and use of KMC are limited. A 2012 evaluation of progress in KMC implementation in Malawi found that only 36% of the facilities assessed had integrated KMC into routine practice and none demonstrated sustainable practice [5]. Lack of documentation and poor record–keeping was found to be widespread and limited the ability of the evaluation to assess other aspects, such as the extent and quality of KMC practice [5]. The 2014 Emergency Obstetrics and Newborn Care (EmONC) survey provides a unique opportunity to address this information gap. The purpose of this paper is to assess the readiness of hospitals in Malawi to provide facility–based KMC and documentation of KMC service delivery.

## METHODOLOGY

### Study setting

Malawi is a small, land–locked country located in Southern Africa with an estimated population of 15.8 million [10]. Administratively, Malawi is organized into five zones (North, Central East, Central West, South East and South West) and 29 districts. Formal health care services are primarily provided by two main agencies: the government, through the Ministry of Health (MOH), operates about 60% of health facilities and the Christian Health Association of Malawi (CHAM) operates an estimated 39%. There is a small contribution from the private–for–profit health sector. Health services are provided at three levels: primary, secondary and tertiary. At primary level, services are delivered through rural hospitals, health centres, health posts, outreach clinics and also through community health initiatives. District and CHAM hospitals provide secondary level health care services to back up the activities of the primary level while central hospitals provide tertiary level and specialized services. At the time of the study, maternal and newborn health services for Malawi’s 29 districts were provided through 87 hospitals and 468 health centres.

### Data source

In 2014, the Ministry of Health in Malawi conducted a nationwide assessment of EmONC services [11]. The sample included 365 public and private health facilities, covering all 87 hospitals and a 60% random sample of the 464 health centres with maternity services. Health facilities that did not offer maternal and newborn health (MNH) services were not included in the sampling frame. Convenience sampling was used to select providers and cases for review within each selected facility.

Data were collected using a structured questionnaire comprised of 10 modules, adapted from the generic modules developed by Averting Maternal Death and Disability (AMDD) [12]. Save the Children worked with AMDD and other stakeholders in Malawi to include additional questions related to KMC for six of the modules (Module 1: Identification of facility and infrastructure; Module 2: Human Resources; Module 3: Essential drugs, equipment and supplies; Module 4: Facility case summary; Module 5: EmONC signal functions; and Module 7: Provider knowledge and competency for maternal newborn care). The modules with the additional KMC questions are available upon request from the authors.

Data were collected from September 23 – October 17, 2014, by 20 teams of three members, all of whom had a clinical background (nursing, midwifery or clinical medicine). Data collectors received five days of training covering the survey tools, research ethics and interview techniques and including field visits and role plays for practice. Quality assurance of data collection was conducted by a supervisor assigned to each team supplemented with a core survey support team comprised of representatives from the MOH, AMDD, Save the Children International, University of Malawi College of Medicine, Medical and Nurses and Midwives Council of Malawi. Double data entry for EmONC data was conducted in CSPro 5.0 and cleaned data files were exported to Stata 12.1 for analysis.

### Analysis of KMC readiness and operational status

Our analysis focused on the 87 hospitals, all of which provide inpatient maternity services and are expected to include facility–based KMC services according to the MoH national guidelines. We used the WHO Service Availability and Readiness Assessment (SARA) domains [13] to identify tracer items for KMC service readiness (staffing & guidelines, equipment & infrastructure, diagnostics, and medicines & commodities) and added a domain for documentation of KMC services provided (**Table 1**). We used standard international definitions of KMC and informal consultations with clinicians to select a list of tracer items that would be needed to implement KMC per the Malawi 2009 guidelines. Levels of KMC service delivery were quantified using data extracted from a 12–month register review (September 2013 – August 2014) and a KMC initiation rate was calculated for each facility by dividing the reported number of babies initiated on KMC by the number of live births at each facility. We defined three levels of KMC readiness (basic, expanded and full) and two levels of KMC operational status (basic readiness plus documentation of KMC services provided); refer to **Table 2** for definitions. Three tracer items were considered essential for provision of basic KMC services: defined space for KMC; at least one staff member reported to provide KMC services and a functional infant weighing scale. We classified the KMC initiation rate into two levels: minimum defined as one or more newborns initiated on KMC per 100 reported live births and routine defined as five or more newborns initiated on KMC per 100 reported live births. Data on the expected number of babies born weighing 2000 g or less and eligible for KMC are limited in Malawi; one recent study reported that 43% of all babies born low birth weight (<2500 g) were <2000 g, which given Malawi’s estimated LBW rate of 13% suggests that around 6% of all live births would be eligible for KMC assuming the ≤2000 g cut–off [14,15]. Results were disaggregated by type of hospital (central, district, community and other).

**Table 1:** Kangaroo Mother Care (KMC) service readiness items captured in 2014 Emergency Obstetric and Newborn Care (EmONC) survey

Domain	Tracer items
**Staffing & guidelines**	Guidelines/protocols for KMC
	Staff providing KMC (any availability)
	Staff providing KMC available 24/7
**Equipment & infrastructure**	Defined space for KMC (separate room or in postnatal area)
	Designated beds for KMC (one or more)
**Diagnostics**	Functional infant weighing scale in delivery and/or postnatal ward
**Medicines and commodities**	Caps/hats for newborns in delivery area
	Linens/blankets for newborns in postnatal area
**Documentation**	KMC register in use
	KMC register complete and up–to–date

**Table 2:** Definitions of indicators for Kangaroo Mother care (KMC) service readiness and KMC operational status

Indicator	Definition
**KMC service readiness:**	**Percentage of hospitals reporting inpatient KMC that have the following:**
**Basic**	1) defined space for KMC
	2) at least one staff providing KMC
	3) functional infant weighing scale
**Expanded**	Basic (*1–3*), plus:
	4) KMC guidelines/protocols available
	5) KMC register available and in use
**Full**	Expanded (*1–5*), plus:
	6) caps/hats for newborn
	7) linens/blankets for newborns
**KMC service operational status:**	**Percentage of hospitals reporting inpatient KMC who have the basic KMC elements (*1–3*), and documentation of:**
**Minimum**	≥1 KMC case initiated/100 live births in last 12 months
**Routine**	≥5 KMC case initiated/100 live births in last 12 months

### Ethical considerations

Ethical approval for the study was granted by the National Health Sciences Research Committee (NHSRC) of Malawi. The survey was led by the MOH, with technical and financial support from AMDD, Save the Children International, WHO, USAID, UNFPA, and UNICEF. Permission to conduct data collection at the facility was granted by the in–charge at each facility and individual oral consent was obtained from all individuals interviewed.

## RESULTS

Data were available for all 87 hospitals, of which 4 were central hospitals, 23 were district hospitals, 33 were community hospitals and 27 were categorized as “other”, which mainly comprised private for–profit hospitals and hospitals operated by Christian Health Association of Malawi (CHAM).

**Figure 1:**
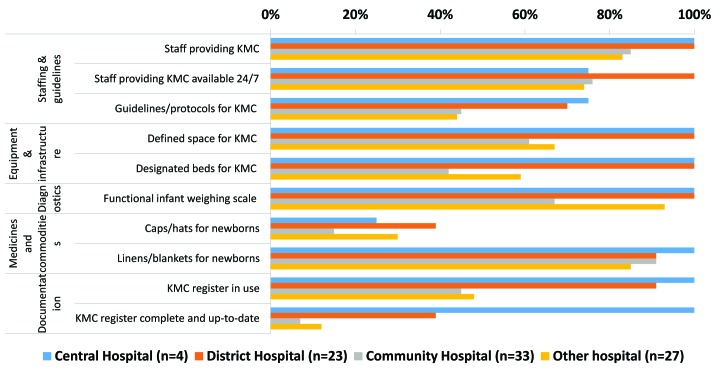
Availability of Kangaroo Mother Care (KMC) tracer items by domain and type of hospital, Malawi Emergency Obstetric and Newborn Care (EmONC) survey 2014.

Most hospitals (79%; 69/87) reported providing inpatient KMC services (range 67% of community hospitals to 100% of central and district hospitals). **Figure 1** shows the availability of KMC tracer items by hospital type. All central and district hospitals had staff for KMC, a defined space and a functional infant weighing scale, compared to two–thirds of other hospitals and less than one–third of community hospitals. Availability of KMC guidelines, caps and hats for newborns were consistently low, even at central and district level hospitals. KMC registers were missing in more than half of community and other hospitals, and few facilities outside of the four central hospitals had up–to–date and complete KMC registers.

**Figure 2:**
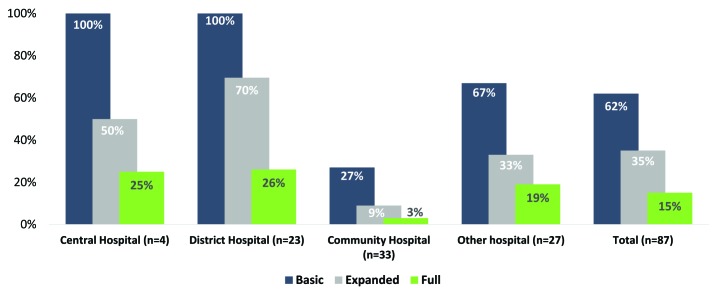
Kangaroo Mother Care (KMC) readiness among hospitals by hospital type, Malawi Emergency Obstetric and Newborn Care (EmONC) survey 2014.

Sixty–two percent of hospitals (54/87) met the basic definition of readiness (staff, space for KMC and functional infant weighing scale) and 35% (30/87) met the expanded definition of readiness (guidelines, staff, space, scale and register in use) (**Figure 2**). Thirteen hospitals (15%) had all KMC tracer items. Community and other hospitals had the lowest levels of readiness.

The 12–month register review yielded a total of 8330 cases initiated on KMC and 211 240 live births across all 87 hospitals, for an overall KMC initiation rate of 3.9/100 live births. More than 80% (84%; 73/87) of hospitals reported providing KMC in the last three months, but just 61% (53/87) had documented cases of KMC services in the 12 months before the survey. Among facilities reporting any KMC cases, the KMC initiation rates ranged from 0.6 cases/100 live births to 17.4 cases/100 live births. Levels of KMC initiation were highest at the central hospitals; of the four central hospitals, three had KMC initiation rates ≥5/100 live births, while one had low levels of KMC initiation (1.8/100 live births). While most (96%) of the district hospitals had KMC initiation rates ≥1/100 live births, 22% had KMC initiation rates ≥5/100 live births. One third of other hospitals and 18% of community hospitals had KMC initiation rates of ≥1/100 live births. In total, 15 of the 87 hospitals recorded KMC initiation rates ≥5 cases/100 live births.

**Figure 3:**
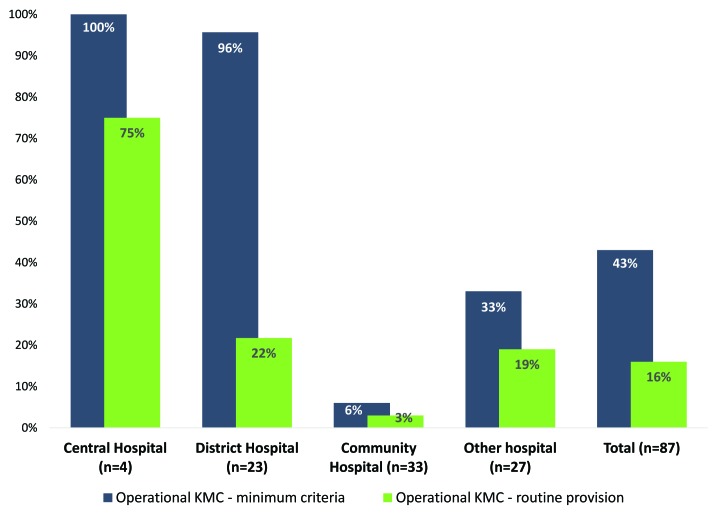
Kangaroo Mother Care (KMC) operational status by hospital type, Malawi Emergency Obstetric and Newborn Care (EmONC) survey 2014

**Figure 4:**
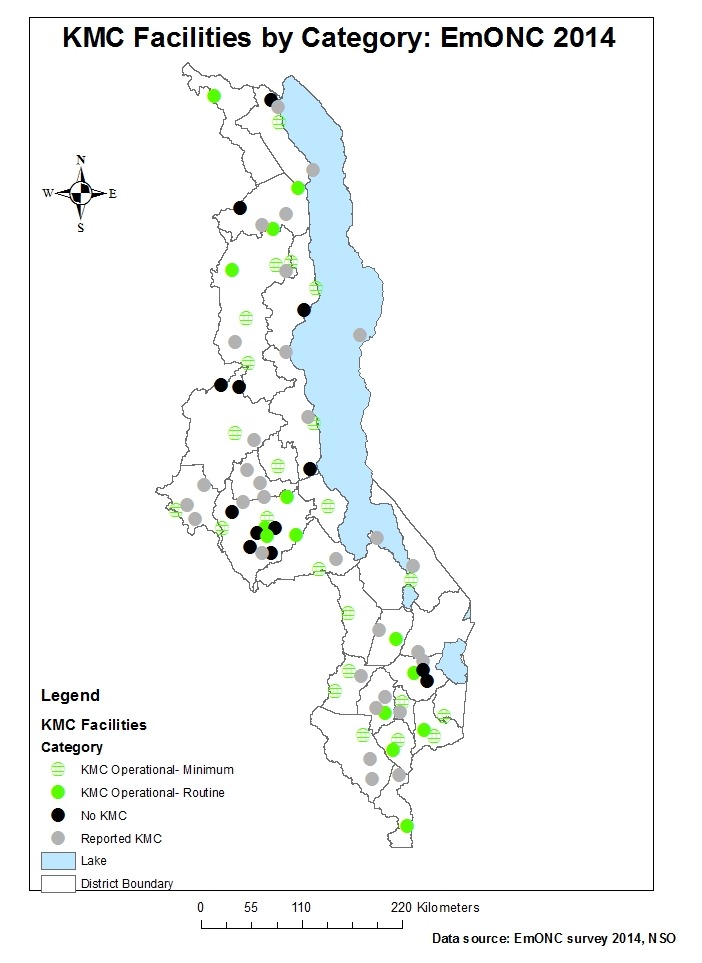
Map of Malawi showing distribution of hospitals by operational status of Kangaroo Mother Care (KMC) services, Malawi Emergency Obstetric and Newborn Care (EmONC) survey 2014.

Less than half of the hospitals (43%; 37/87) met criteria for KMC operational status at minimum levels (**Figure 3**). All central and nearly all district hospitals (96%) met criteria for minimum operational KMC, compared to 33% of other hospitals and 6% of community hospitals (**Figure 4**). Fourteen of Malawi’s 87 hospitals (16%) met criteria for KMC operational status at routine levels (≥5/100 live births) (**Figure 4**).

## DISCUSSION

Malawi has been systematically scaling up KMC services since the intervention was introduced in 1999 and has set ambitious targets for coverage of KMC as part of its ENAP plan, aiming for 75% of eligible newborns to receive KMC by 2020. However, our analysis of the 2014 EmONC needs assessment in Malawi, the first such survey to capture detailed information on KMC services at national level, suggest that much more needs to be done if Malawi is to reach its goals. Readiness of hospitals to provide KMC was just a fraction of reported service availability. While nearly 80% of hospitals reported providing KMC services, less than two–thirds of hospitals had the minimum tracer items and only one in six had all tracer items. Our study also found poor documentation of KMC services and low levels of KMC initiation, apart from a few hospitals with well–established KMC services. Overall, just 14 of Malawi’s 87 hospitals met the criteria for basic readiness and demonstrated providing KMC services routinely (at least 5 cases initiated on KMC per 100 live births).

Readiness to provide KMC services was limited primarily by lack of guidelines, caps/hats for newborns and service documentation. Having national guidelines in place at health facilities and health workers trained to use them in addition to emphasis on skills strengthening through mentorship sessions can help ensure standardization of service provision. At present, provision of caps/hats is not standard practice as mothers are expected to bring their own to the facility. Availability of caps/hats, which support thermal care, is especially important for women experiencing preterm birth who may not be able to provide their own. Our study found that KMC documentation continues to be a challenge, particularly for district and other hospital types. At the time of the study, a register and monthly report form to track KMC services was developed, but the tools were not nationally endorsed and dissemination was ad hoc. Consequently, routine service data for KMC were limited and incomplete.

Readiness and documentation of service provision were lowest among community level hospitals. Many community hospitals lacked designated beds for KMC and basic equipment such as a functional infant scale and fewer than one in five initiated at least one case per 100 live births on KMC. In some districts, inpatient KMC services have not scaled up to community level hospitals, which often lack the infrastructure and human resource capacity to manage all units as a hospital. The common practice has been to initiate babies on KMC and refer them to facilities with inpatient KMC or to community for ambulatory KMC, leading to documentation challenges as these referrals are often not recorded.

Our study found stark differences between reported availability of KMC, readiness to provide KMC, and documented KMC service provision. Facility reports of service availability overestimated the level of KMC services, especially when the expanded or full set of tracer items were applied. The recommended global ENAP process indicator for KMC is the proportion of facilities in which a space is identified for KMC and where staff have received training in KMC in the last two years [16]. The ENAP process indicator is similar to our definition of basic readiness (staffing, defined space, and a scale), which was met by most facilities. While the EmONC study tool gathered information about staff availability to provide KMC and not KMC training directly, our results suggest that reporting on the ENAP process indicator would overestimate the availability of KMC services in Malawi. The periodic capture and use of several additional tracer items will provide a better picture of facility readiness to provide KMC services. Work is under way to develop a standard list of tracer items by KMC experts as part of the ENAP indicator development and validation process [16]. We also assessed whether facilities had ‘operational’ KMC, by combining basic criteria of readiness with levels of documented KMC service provision, and found that less than half of hospitals met the most minimum level of operational status, largely due to low levels of documented KMC initiation. This suggests that capturing readiness alone, as measured by availability of tracer items, is also prone to exaggerate service availability. Measures of service delivery should be captured alongside readiness where possible to obtain a clearer picture of how operational KMC is in a given facility.

While our findings suggest that we can improve the assessment of KMC service availability through better measurement of key inputs (readiness tracer items) and service delivery (operational status), understanding the strength and quality of KMC implementation at the facility and patient level will also be critical to achieving impact. As national surveys are not necessarily appropriate for gathering information on quality of care, supplementary studies and quality initiatives will be necessary for a complete picture of KMC service provision. Reporting on KMC availability, readiness, and operational status are necessary, but not sufficient indicators of KMC provision. Indeed, the presence of staff, supplies, and space for KMC is a prerequisite for quality implementation of KMC; but assessment of the quality of key components of KMC—skin–to–skin care and exclusive breastfeeding—is also needed to achieve meaningful process evaluation and scale–up of this life–saving intervention.

Building on momentum from the launch of ENAP, Malawi is investing in efforts to strengthen quality of newborn care services for small and sick babies, including KMC, and to improve documentation and reporting. The MoH is collaborating with partners, including Save the Children, MaiKhanda and others, to create an institutionalized mechanism for quality improvement of services for small and sick newborns through strengthening system building blocks such as leadership, financing, staffing, essential drugs and supplies, information systems, and ownership and partnership. The initiative aims at integrating functional small and sick newborn units, capacity building through mentorship and coaching, documentation and sharing of learning in all central and district hospitals in Malawi. Efforts such as these, in addition to Malawi’s participation in the KMC Acceleration Partnership Community of Practice, provide an opportunity to improve quality and strength of implementation of KMC at the facility and patient level.

Since the EmONC study was completed, the Malawi Reproductive Health Directorate and Central Monitoring and Evaluation Department (CMED), have taken important steps to address the poor documentation and reporting of KMC [17]. In October 2015, the MoH began rolling out a national routine reporting system for KMC, which includes a simplified, user–friendly KMC register and reporting tool designed to generate a set of core indicators for tracking KMC implementation and making clinical and management decisions to improve the quality of KMC services. Data are entered at the district level into the DHIS2 (Malawi’s health information system platform) and the core indicators are calculated automatically. Ongoing efforts are needed to strengthen the timeliness, completeness and quality of the data and encourage regular use at facility, district and national levels.

### Limitations

This study has some important limitations. We looked at the availability of selected tracer items for KMC services; other items required to provide quality care for small babies, such as nasal gastric feeding tubes, cups and spoons for feeding, and patient monitoring charts, were not captured. At the time the Malawi EmONC survey tools were being developed, consensus regarding what tracer items should be captured for KMC was not available. The preparation of such a standardized list, as currently in process through the ENAP metrics working group, will improve such assessments in future. We relied on KMC registers to look for evidence of KMC service delivery. However, the availability and completeness of register data are often low, as was seen in this assessment. Some facilities may have been providing KMC services without using the registers, which could underestimate the level of KMC services being provided. Further, we were unable to assess the quality of the register data, and it is unclear how data quality issues would affect the results. We attempted to assess ‘operational’ KMC, combining measures of basic readiness with documentation of service delivery. However, EmONC surveys, like most facility assessments, rarely include an observational component and are unable to determine important aspects of the quality of care provided, and as such our measures of operational KMC do not take this into account.

## CONCLUSIONS

We found large differences between reported levels of KMC services and documented levels of KMC readiness and service provision among hospitals in Malawi. While many hospitals met the basic criteria for KMC readiness, few had most or all tracer items. Levels of documented KMC initiation were much lower than needed to achieve high coverage of KMC for preterm and LBW babies in Malawi.

We recommend that, when feasible, facility assessments of services, such as KMC, include record reviews to better estimate service availability and delivery. Further efforts to strengthen capacity of Malawian hospitals to deliver KMC are needed, particularly for district, community and other hospitals. Such efforts should include routine reviews of KMC data by facility for gaps and ensuring basic items are available to hospitals providing inpatient KMC. Regular assessment of levels of KMC service delivery through the existing DHIS2 are required to identify under–performing facilities and provide further support and supervision.
